# aPKC Phosphorylation of HDAC6 Results in Increased Deacetylation Activity

**DOI:** 10.1371/journal.pone.0123191

**Published:** 2015-04-10

**Authors:** Yifeng Du, Michael L. Seibenhener, Jin Yan, Jianxiong Jiang, Michael C. Wooten

**Affiliations:** 1 Department of Biological Sciences, Cellular and Molecular Biosciences Program, Auburn University, AL, 36849, United States of America; 2 Graduate Center for Toxicology, Markey Cancer Center, University of Kentucky College of Medicine, Lexington, KY, 40536, United States of America; 3 James L. Winkle College of Pharmacy, University of Cincinnati, Cincinnati, OH, 45267, United States of America; Oregon State University, UNITED STATES

## Abstract

The Class II histone deacetylase, HDAC6, has been shown to be involved in cell motility, aggresome formation and mitochondria transport. HDAC6 deacetylase activity regulates α-tubulin acetylation levels and thus plays a critical role in these processes. In turn, HDAC6 activity can be regulated by interaction with various proteins including multiple kinases. Kinase mediated phosphorylation of HDAC6 can lead to either increased or reduced activity. Our previous research has shown that sequestosome1/p62 (SQSTM1/p62) interacts with HDAC6 and regulates its activity. As SQSTM1/p62 is a scaffolding protein known to interact directly with the zeta isoform of Protein Kinase C (PKCζ), we sought to examine if HDAC6 could be a substrate for PKCζ phosphorylation and if so, how its activity might be regulated. Our data demonstrate that HDAC6 is not only present in a protein complex with PKCζ but can also be phosphorylated by PKCζ. We also show that specific phosphorylation of HDAC6 by PKCζ increases HDAC6 deacetylase activity resulting in reduced acetylated tubulin levels. Our findings provide novel insight into the molecular mechanism by which HDAC6, PKCζ and SQSTM1/p62 function together in protein aggregate clearance. These results also highlight a new research direction which may prove fruitful for understanding the underlying cause of several neurodegenerative diseases.

## Introduction

The class II histone deacetylase 6 (HDAC6) has been found to be associated with diverse cellular processes. The deacetylation of multiple targets such as tubulin, hsp90, cortactin, and histone by HDAC6 is well documented [1, 2]. The best characterized of these interactions is with the α-tubulin subunit of microtubules. HDAC6’s control of the acetylation state of these subunits is an essential component of cell migration and motility [3, 4], aggresome clearance [5], mitochondria transport [6] and dynein associated retrograde transport along microtubules [7]. While it is known that deacetylation of α-tubulin influences the functional aspects of these processes, it is only recently that the exact modes of HDAC6 regulation are being elucidated. HDAC6 is predominantly localized to the cytoplasm where interaction with direct or indirect binding partners results in regulation of HDAC6’s activity [8, 9]. For example, direct binding to tau, a microtubule associated stabilizing protein, directly inhibits HDAC6 deacetylase activity leading to impairment of autophagy [10].

In addition to protein-protein interactions affecting its activity, HDAC6 has also been shown to be regulated by post-translational modifications such as phosphorylation. A number of different kinases have been implicated as phosphorylation agents. An EGFR mediated phosphorylation pathway leads to reduced deacetylase activity of HDAC6 resulting in negatively regulated EGFR endocytosis and degradation [11]. Conversely, HDAC6 phosphorylation mediated by G protein-coupled receptor kinase 2 (GRK2) and casein kinase 2 (CK2) regulate cell motility and aggresome formation by increasing the deacetylase activity of HDAC6 [4, 5]. HDAC6 contains two deacetylase catalytic domains, DD1 and DD2. Interestingly, studies have indicated that phosphorylation sites potentially exist both within the two catalytic domains and outside of them [11 – 13], suggesting the possibility for indirect regulation of HDAC6’s catalytic activity through allosteric conformation changes [3 – 6].

Given the multifaceted role played by HDAC6 in cellular function, understanding kinase-mediated regulation of HDAC6 is important. PKCs can serve as important cytoskeleton regulators involved in cell polarization, directional sensing, and cell motility [14]. The classical PKC isoform PKCα recruits and activates HDAC6 by phosphorylation. This interaction modulates HDAC6’s deacetylation of β-catenin, enhancing its nuclear translocation and promoter binding [15, 16]. Atypical PKC (aPKC) is essential for the regulation of cell polarization, cell motility and migration of macrophages [14]. Recent investigations suggest that the aPKC-aurora A-NDEL1 pathway is crucial for the regulation of microtubule dynamics [17]. Inhibition of aPKC prevents the activation of HDAC6 and stabilizes primary cilia [18]. In addition, two cytosolic aPKC isoforms have been shown to phosphorylate AurA, which then targets HDAC6, stimulating tubulin deacetylation in primary cilia [19]. However, to date, there is little evidence indicating any direct association between aPKC and HDAC6. Recently our laboratory reported that the multimeric scaffolding protein SQSTM1/p62 binds to HDAC6, negatively regulating its deacetylase activity and affecting microtubule network equilibrium [20]. SQSTM1/p62 was originally isolated and characterized as an aPKC binding protein [21] with roles defined in ubiquitin binding [22] and cytoplasmic aggregate formation [23, 24]. Thus we reasoned that the SQSTM1/p62 binding partner PKCζ could regulate HDAC6 deacetylase activity by direct phosphorylation of the HDAC6 protein. Here we show that HDAC6 is a substrate for PKCζ phosphorylation and that the deacetylase activity of HDAC6 is induced by PKCζ specific phosphorylation.

## Materials and Methods

### Cell Culture and Transfection

Human embryonic kidney (HEK) 293 cells and Mouse Embryonic Fibroblast (MEF) from the American Type Culture Collection were grown as described previously [25]. Transfections were achieved using jetPRIME DNA Transfection Reagent (Polyplus Transfection, VWR International) following the manufacturer’s directions. Cells were grown in DMEM supplemented with 10% Heat-inactivated Fetal Calf Serum (Atlanta Biologicals, Atlanta, GA) and antibiotic/antimyotic solution (Sigma-Aldrich, St. Louis, MO) at 37°C with high humidity and 5% CO_2_ environment.

### Antibodies and Reagents

Antibodies for FLAG, MYC, and HA tags were from Abcam (Cambridge, MA). Phospho specific antibodies for serine and threonine residues were from Sigma-Aldrich (St. Louis, MO). Antibodies for α-tubulin and acetyl-tubulin were also from Sigma-Aldrich (St. Louis, MO). Texas Red, Cy5 and Oregon Green fluorescent secondary antibodies were from Life Technologies (Carlsbad, CA). aPKC pseudosubstrate inhibitor and Tubucin were purchased from EMD Biosciences (San Diego, CA). Purifed PKCζ was from EMD Biosciences.

### Immunoprecipitation and Western Blot Analysis

HEK cells were lysed on tissue culture plates on ice with Triton Lysis Buffer (TLB, 50mM Tris pH 7.5, 150mM NaCl, 10mM NaF, 1mM Na_3_VO_4_, 0.5% Triton X-100, 10μg/ml leupeptin, 10μg/ml aprotinin and 1mM PMSF). Protein was determined by Bradford Assay and equal amount of whole cell lysate (500μg) was incubated with 3μg of appropriate primary antibody for 3 hours at 4°C followed by the addition of agarose beads (50 μl) specific for the primary antibody. Immunoprecipitation was allowed to rotate overnight at 4°C. The immunoprecipitates were then washed five times with TLB. Proteins were released from agarose beads by boiling 2 minutes in SDS-PAGE sample buffer and then separated by 10% SDS-PAGE followed by transfer to nitrocellulose membrane and Western blot analysis with the corresponding antibodies. ECL solution (GE Healthcare, Piscataway, NJ) was used to visualize proteins.

### Immunoprecipitation Activity Assay

FLAG-tagged HDAC6 was expressed in HEK cells and immunoprecipitated using anti-FLAG antibody as described above in Activity Lysis Buffer (150 mM NaCl, 50 mM Tris, 0.1% TX-100, 2 mM EDTA, 1 mM EGTA, 1 mM PMSF, 25 μg/ml leupeptin, 25 μg/ml aprotinin, 1 mM Na_3_VO_4_, 2.5 mg/ml PnPP). Washing of the immunoprecipitate was with Activity Wash Buffer (35mM Tris, 150mM NaCl, 15mM MgCl_2_, 1mM MnCl_2_, 0.5mM EGTA, 0.1% TX100, 25μg/ml leupeptin, 25μg/ml aprotinin). Following the final wash, 0.5μg active recombinant aPKCζ enzyme (EMD Biosciences, San Diego, CA) was added in Activity Assay Buffer (35mM Tris, 15mM MgCl_2_, 1mM MnCl_2_, 0.5mM EGTA, 1mM Na_3_VO_4_) to the pelleted agarose beads along with 5μCi [γ-^32^P]-ATP (Perkin-Elmer, Akron, OH) and 10μM cold ATP. Phosphorylation reactions were allowed to react for 30 minutes at 30°C with constant agitation. Reactions were stopped by the addition of equal volume 2X Laemmli Sample Buffer and labeled immunoprecipitates were resolved on 10% SDS-PAGE gels. Gels were then fixed in 45% methanol/10% Acetic acid prior to drying and exposure to x-ray film.

### Immunofluorescence

MEF cells were grown on poly-D-lysine: rat tail collagen (3:1) coated coverslips in 24 well plates. For immunofluorescence, MEF cells were washed with phosphate-buffered saline (PBS), followed by fixation in freshly made warm 4% paraformaldehyde/PBS. Following fixation, permeabilization was in 0.1% Triton X-100/PBS for 15 min prior to blocking with 3% milk/PBS for 1 hour at room temperature. Endogenous proteins were detected with specific antibody (1:100) in block overnight at 4°C. Following removal of primary antibody by 3 times washing with PBS, fluorescently tagged secondary antibody (1:400) was added in block for 1 hour at room temperature. Cells were washed a total of 5 times with PBS prior to mounting to slides. Where indicated, cells were transfected with construct for specific protein expression as described above. Fluorescently tagged proteins were analyzed with 60X oil immersion on a Nikon A1/T1 confocal microscope and images processed employing NIS Elements Software AR 3.22.13 [Build 730](Nikon).

### Statistical Analysis

For each experimental condition, a minimum of 25 individual cells were photographed and intensity values measured using NIS Elements Software AR 3.22.13 [Build 730] (Nikon). Means, standard errors, and *t*-tests were calculated manually [26]. *T*-test statistics were calculated under the assumption of unequal group variances with one-tailed *p*-values. *P*-values less than 0.05 were considered significant.

## Results and Discussion

**Fig 1:**
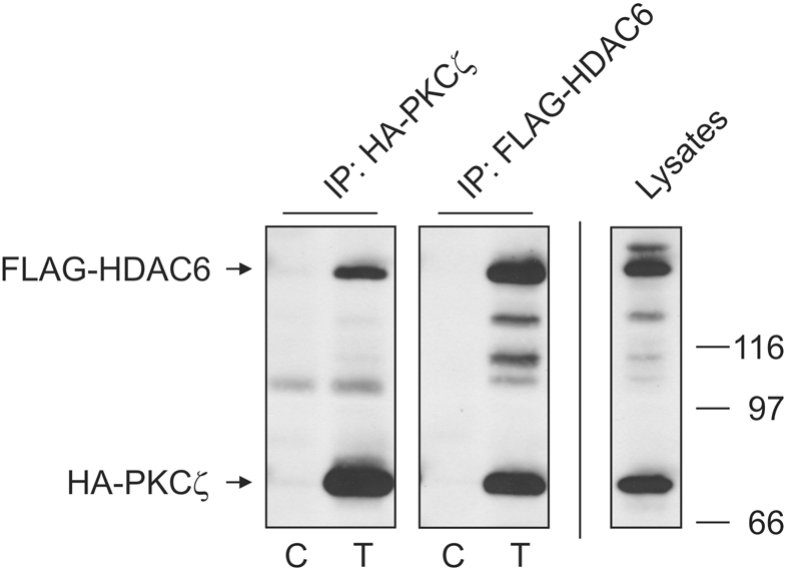
Atypical PKCζ exists in a complex with HDAC6. HA-PKCζ and FLAG-HDAC6 were transfected into HEK cells and reciprocal immunoprecipitations performed using antibodies for each tag as described in Materials and Methods. Control lanes (C) were transfected with empty plasmid while sample lanes (T) were transfected with both HA-PKCζ and FLAG-HDAC6. 40 μg of whole cell lystate (Lysate) was loaded to show construct expression in transfected cells. Co-precipitating bands were detected by SDS-PAGE and Western blot with tag-specific antibodies. Results are indicative of 3 independent experiments.

Protein interactions are essential for cellular functions. Many of these interactions are part of signaling pathways that regulate protein activities. Phosphorylation is an important signaling mechanism resulting in modulation of a protein’s activity within a signaling cascade. We have previously shown that the activity of the histone deacetylase protein HDAC6 can be modulated by the scaffolding protein SQSTM1/p62 [20]. SQSTM1/p62, in complex with atypical protein kinase C (aPKC), has also been implicated as an important regulatory module controlling the activation of NF-κB by phosphorylation in stroma cells of cancer patients [27]. The aPKC, PKCζ, can positively affect NF-κB at the transcriptional level by specific phosphorylation of its p65 subunit [28] while also displaying a negative regulatory role in IL-6 production in an NF-κB independent manner [29, 30]. It is well recognized that HDAC6 activity is regulated by phosphorylation. This regulation in turn has direct impact on cell motility [3, 31] and clearance of cellular aggresomes [5]. Based on these reports and our previous work, we sought to determine if an aPKC, specifically PKCζ, could phosphorylate HDAC6 and regulate its deacetylase activity. Using tagged protein constructs, we exogenously expressed FLAG-HDAC6 and HA-PKCζ in HEK cells and examined their interaction by immunoprecipitation (Fig 1). By immunoprecipitating either tagged protein, we were able to visualize the other as part of the captured immune complex. While not proving direct interaction, these results do indicate that the two proteins can exist as part of the same complex. This raises the possibility that HDAC6 could be regulated by the kinase activity of PKCζ.

**Fig 2:**
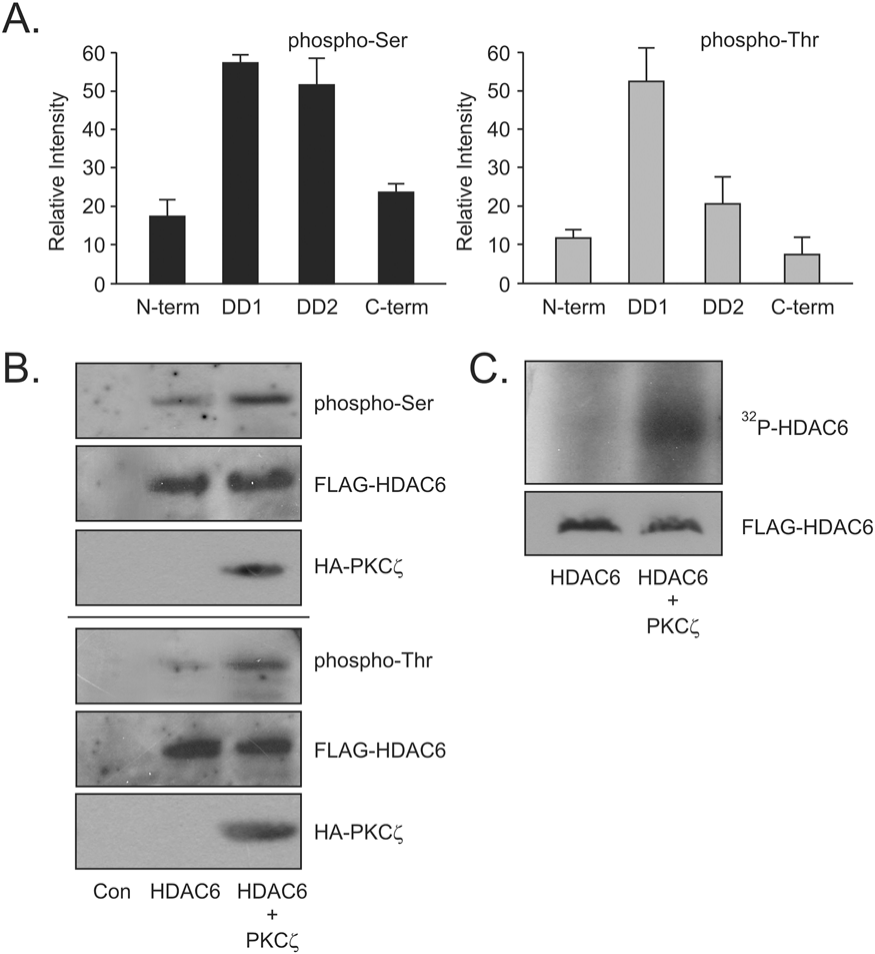
Atypical PKCζ phosphorylates HDAC6. (**A**) GST-deletion constructs of HDAC6 were expressed in HEK cells and captured by GST-pulldown assay. Purified PKCζ enzyme was used to catalyze an *in-vitro* phosphorylation assay using the GST-deletion constructs as substrate. Phosphorylation of each construct was assessed using SDS-PAGE and phospho-specific antibodies for phospho-Serine and phospho-Threonine. Relative changes in phosphorylation were measured by densitometry and analyzed using UN-SCAN-IT 6.1 Software (Orem, UT). (**B**) FLAG-HDAC6 was expressed in HEK cells either with or without HA-PKCζ expression. *In vivo* phosphorylation increases were detected using phospho-specific antibodies. (**C**) FLAG-HDAC6 was expressed in HEK cells and immunoprecipitated with anti-FLAG antibody. The immunoprecipitate was used as a substrate in an immune-complex kinase assay with purified PKCζ enzyme as the catalyst. Changes in phosphorylation were detected by incorporation of ^32^P-ATP and autoradiography. All results are indicative of 3 independent experiments.

The DD1 and DD2 domains of HDAC6 have been shown to be critical to the deacetylase activity of the protein [32]. Some studies have suggested that both are required for tubulin deacetylation [32], whereas others found that only DD2 was essential [12, 13]. Phosphorylation of the Tyrosine570 residue in the DD2 domain of HDAC6 results in reduced deacetylase activity, leading to increased acetylation of α-tubulin and subsequent decrease in receptor trafficking along the microtubule [11]. Phosphorylation sites that affect deacetylase activity have also been identified outside of these two catalytic domains [3 – 5]. Phosphorylation at Serine458, located between the DD1 and DD2 domains, by CK2 upregulates the deacetylase activity of HDAC6 resulting in the loading of misfolded proteins to the dynein motor protein for transport to aggresomes [5]. As PKCζ and HDAC6 can exist in a protein complex, we wished to see if HDAC6 was phosphorylated by PKCζ. GST-HDAC6 domain expression constructs were used to generate exogenous protein that was harvested from HEK cells and used as a substrate for phosphorylation in an *in vitro* kinase assay with purified PKCζ. Phosphorylation state was determined by Western blot using antibodies specific for phospho-serine and phospho-threonine residues. GST-HDAC6-N-terminal (1–85) and GST-HDAC6-C-terminal (825–1149) showed only modest phosphorylation above basal levels. However, PKCζ phosphorylated both serine and threonine residue(s) in DD1 while also phosphorylating predominantly serine residue(s) in DD2 (Fig 2A). Thus, in an *in vitro* assay, PKCζ was successful in phosphorylating HDAC6. Full length FLAG-HDAC6 was then expressed in HEK cells either with or without HA-PKCζ. When FLAG-HDAC6 was examined as a substrate for phosphorylation with phospho-specific antibodies, it too exhibited an increase in both phospho-serine and phospho-threonine levels when PKCζ was overexpressed above basal levels (Fig 2B) indicating intracellular modification of the phosphorylation state of HDAC6. This increase in phosphorylation of FLAG-HDAC6 was further confirmed in an immune-complex kinase assay where FLAG-HDAC6 was immunoprecipitated and used as the substrate for purified PKCζ γ-^32^P-ATP. An increase in ^32^P- ATP associating with HDAC6 is clearly evident when PKCζ was added to the kinase assay (Fig 2C). Taken together, this data confirmed that HDAC6 was indeed a substrate for PKCζ phosphorylation. It also suggests that PKCζ phosphorylation sites are predominantly in the conserved DD1 and DD2 catalytic regions of HDAC6. Having shown that PKCζ does interact in a complex with HDAC6 resulting in specific phosphorylation of HDAC6, we next wished to address if this phosphorylation event had a regulatory effect on the deacetylation activity of HDAC6.

**Fig 3:**
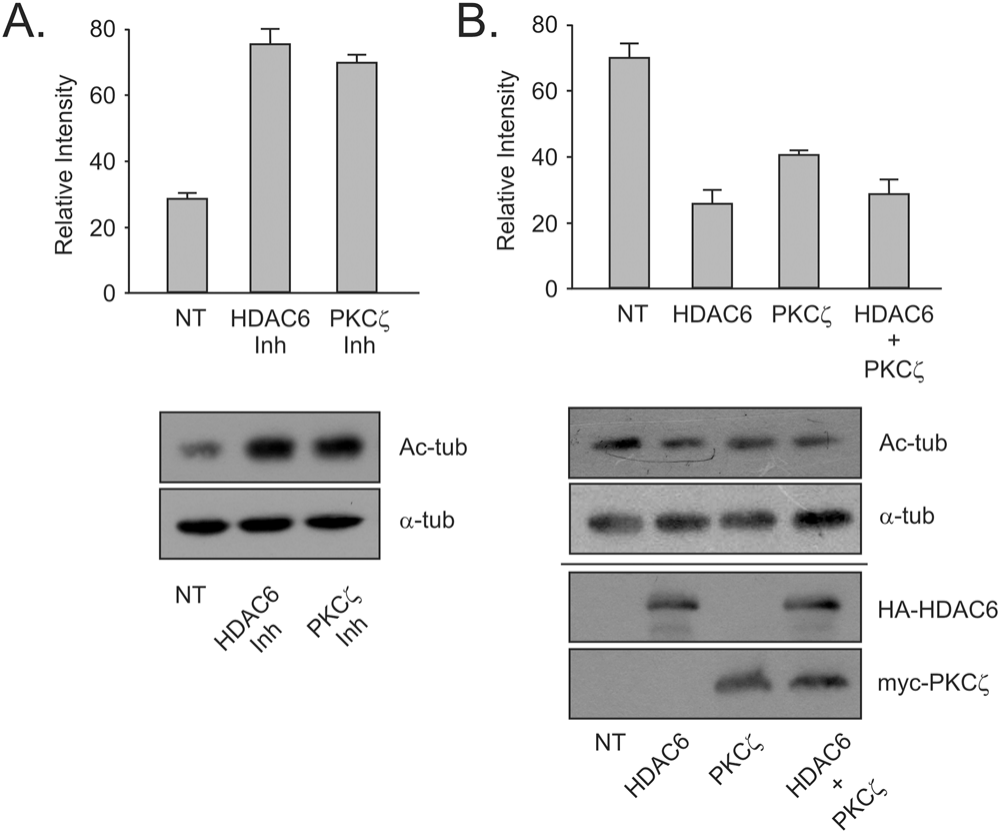
Phosphorylation by atypical PKCζ upregulates the deacetylase activity of HDAC6. (A) MEF cells were treated with HDAC6 inhibitor (tubacin—10μM for 4 hours) or aPKC inhibitor (myristoylated pseudosubstrate—20μM for 16 hours) and HDAC6 endogenous activity was assessed using changes in acetylated-tubulin levels. A total of 40μg whole cell lysate was loaded in each lane. (B) HA-HDAC6 and myc-PKCζ were expressed separately or in concert in HEK cells and acetylated tubulin levels assessed by SDS-PAGE and Western blotting. Expression blots for lysates were loaded with 40μg lysate protein. Results are indicative of 3 independent experiments.

α-Tubulin is a well characterized intracellular substrate of HDAC6 deacetylase activity. Acetylation of α-tubulin at Lys40 is one characteristic of stable microtubules [33, 34] while mistimed deacetylation of microtubules has been linked to neurodegenerative diseases [35]. Using α-tubulin as a test substrate we have previously shown that the aPKC interacting protein SQSTM1/p62 plays a role in regulation of HDAC6 activity [20]. Thus, we reasoned that acetylation of α-tubulin would provide a readout of the phosphorylation effects on HDAC6 by PKCζ. Using this model, we examined what effect inhibition of either HDAC6 or PKCζ activity had on acetylated tubulin levels. Non-transfected mouse embryonic fibroblasts (MEF) cells were treated with either the HDAC6 deacetylase activity specific inhibitor tubacin or myristoylated aPKC pseudosubstrate inhibitor to see effects of inhibition on endogenous protein activity. The increase in acetylated tubulin in cells treated with aPKC inhibitor was comparable to that seen when cells were treated with HDAC6 specific inhibitor. Thus, preventing aPKC specific phosphorylation of HDAC6 had a similar effect on HDAC6 deacetylase activity as seen with specific HDAC6 inhibition (Fig 3A). Combined these results indicate that the kinase activity of PKCζ is capable of upregulating the deacetylase activity of HDAC6.

With the data now indicating that inhibition of aPKC promotes acetylation of α-tubulin due to HDAC6 inactivation, the next step was to demonstrate a reverse effect. We predicted that increasing either PKCζ or HDAC6 would produce lowered tubulin acetylation levels. Increasing the amount of HDAC6 in the cytoplasm of fibroblasts has previously been shown to decrease acetylation of α-tubulin [20]. When HDAC6 was exogenously overexpressed in HEK cells, the expected drop in acetylated tubulin was observed (Fig 3B). Overexpression of PKCζ produced a similar but less drastic decrease in acetylated tubulin. When PKCζ was co-expressed with HDAC6, the levels of acetylated tubulin present in the cells showed a drop comparable to the levels seen with HDAC6 alone (Fig 3B). This outcome was most likely due to the large amount of HDAC6 exogenously expressed in the cells producing a titer that resulted in hiding any extra activation effect shown by co-expression of PKCζ. With tubulin acetylation as a specific readout of HDAC6 activity, we see that increasing levels of PKCζ results in upregulation of the activation state of HDAC6 causing a corresponding reduction in acetylation levels of α-tubulin. This suggests that HDAC6 phosphorylation by PKCζ does result in an increase in deacetylase activity.

**Fig 4:**
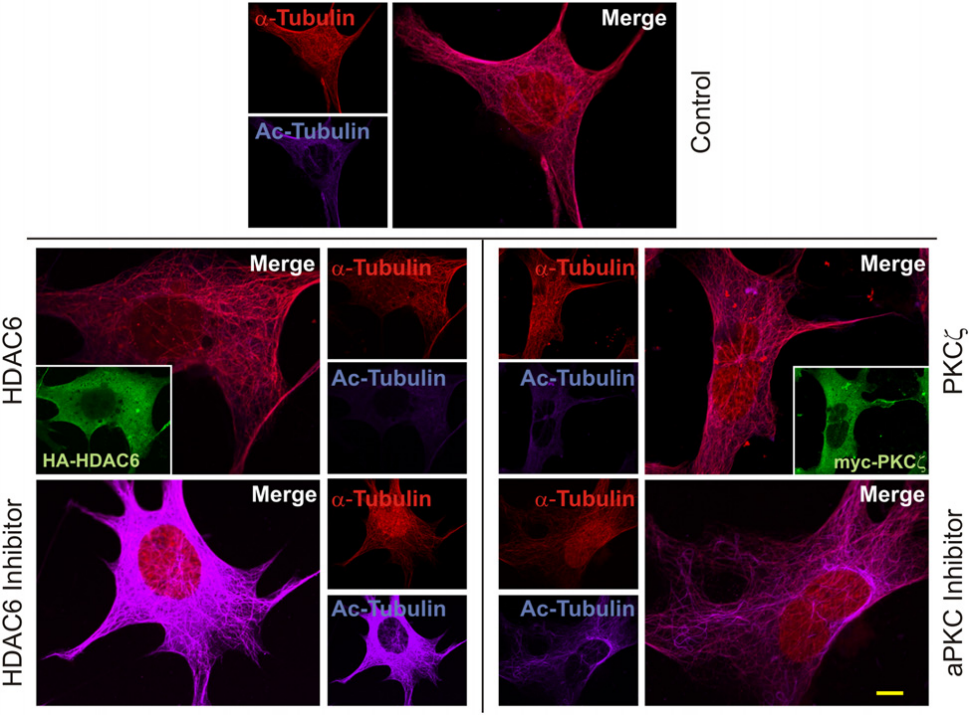
Atypical PKCζ regulates HDAC6 deacetylase activity. Parental MEF cells were transfected to overexpress either HA-HDAC6 or myc-PKCζ or were treated with HDAC6 inhibitor tubacin (10μM for 4 hours) or aPKC inhibitor (myristoylated pseudosubstrate—20μM for 16 hours). Cells were subsequently fixed with 4% paraformaldehyde prior to immunofluorescence staining using α-tubulin antibodies (1:100) detected with Cy5 secondary antibody (1:400) and acetyl-tubulin antibody (1:100) detected with Texas Red secondary antibody (1:400). Texas Red labeled acetyl tubulin was pseudocolored to 400nm using Nikon NIS Elements AR software to visually separate it from Cy5. Inset panels for HA-HDAC6 and myc-PKCζ (HA and myc antibodies used at 1:100) overexpression were detected with Oregon Green secondary antibody (1:400). Images from a minimum of 25 cells each were used for statistical analysis. Scale bar is 10μm.

The impact of inhibition or overexpression of PKCζ and HDAC6 on acetylation of tubulin was further visualized by immunofluorescence. MEF cells were transfected with tagged HDAC6 or treated with tubacin [12]. Both α-tubulin and acetyl-tubulin were identified using specific antibodies and visualized with fluorescent tagged secondary antibodies. All images were produced using the same settings on a Nikon A1/T1 confocal microscope so that comparisons in intensity could be analyzed between different cells. The intensity of the fluorescence from the α-tubulin and acetylated tubulin images were measured using NIS-Elements AR software (Nikon). Intensity values for α-tubulin were normalized across all samples and subtracted from the acetylated tubulin values of the corresponding panels. This allowed the remaining acetylated tubulin fluorescent intensity to be used as a measure of the activity of HDAC6. Control non-transfected and untreated cells were first analyzed for basal levels of α-tubulin acetylation (483.55 +/- 23.38). Overexpression of HDAC6 (seen in inset, Fig 4) resulted in low relative levels of acetylated tubulin in the cell due to increased deacetylase activity upon the substrate (360.67 +/- 14.22; *t*(51) = 4.012, *p* = 0.0004; a representative panel is shown in Fig 4). However, when MEF cells are treated with HDAC6 inhibitor, acetylated tubulin fluorescence intensity levels (1987.77 +/- 196.19; *t*(48) = -9.384, *p*<0.0001) were strikingly higher being approximately 6-fold greater when compared to the HDAC6 overexpressing cells. Similarly, overexpression of PKCζ resulted in low acetylated tubulin intensity values (377.26 +/- 59.49; *t*(52) = 2.519, *p* = 0.011) approximately equal to HDAC6 overexpression. Inhibition of PKCζ activity by pseudosubstrate treatment showed greater intensity of acetylated tubulin (624.91 +/- 35.58; *t*(48) = -4.708, *p* = 0.0001) with approximately a 2-fold increase over exogenous PKCζ expression. This was less than was seen with HDAC6 inhibition. However, it is entirely plausible that PKCζ is not the only kinase that activates HDAC6. Thus, even when PKCζ phosphorylation of HDAC6 is inhibited, HDAC6 deacetylase activity can still function, though obviously at a reduced level. These results do however confirm that PKCζ levels in the cell or the activity state of PKCζ can regulate HDAC6 activity.

Multiple functions of HDAC6 have been well documented in the past decade. It is known to be involved in microtubule dynamics and cellular organization associated with neurodegenerative diseases and cancer [35, 36]. Many kinases have been demonstrated to phosphorylate HDAC6 and regulate its activity. In the light of the results reported herein, we conclude that the aPKC isoform, PKCζ, can phosphorylate HDAC6 and upregulate its deacetylase activity. This finding provides a clue into the molecular mechanism linking HDAC6 activity within normal cells to potential roles in neurodegenerative disease and cancer. Our results indicate that further investigations into the viability of using HDAC6 as a target for treatment of neurological diseases may be warranted.
